# Molecular identification and antibiotic resistance profiling of Salmonella species isolated from chickens in eastern Turkey

**DOI:** 10.1186/s12917-020-02425-0

**Published:** 2020-06-19

**Authors:** Aydogan Arkali, Burhan Çetinkaya

**Affiliations:** 1 Veterinary Control Institute, 23100 Elazig, Turkey; 2 Department of Microbiology, Veterinary Faculty, Firat University, 23100 Elazig, Turkey

**Keywords:** *Salmonella*, Chicken, mPCR characterization, Antibiotic resistance profiling

## Abstract

**Background:**

The aim of this study was to obtain quantitative data about the frequency, genotypic characterization and antibiotic resistance profiling of *Salmonella* agents in chicken flocks located in eastern Turkey.

**Results:**

Feces samples representing at least 20% of the flock area were collected via sock swabs from commercial poultry flocks in the study region in addition to internal organs (liver, spleen, intestine) collected at necropsy of suspected chickens belonging to small family enterprises. The samples were analyzed by conventional bacteriological methods (ISO 6579:2002/A1:2007) for isolation, and genus specific (*invA*) PCR for the identification of *Salmonella* spp. Then, two mPCR were set up to determine *Salmonella* serotypes and genotypic resistance status of the field isolates against ampicillin, tetracycline, trimethoprim-sulfamethoxazole and chloramphenicol antibiotics. In the PCR analysis of the suspected colonies, 98.5% were confirmed as *Salmonella* spp., and, the most prevalent serotype was identified as *S*. Infantis with the proportion of 26.6% (17/64), followed by *S.* Enteritidis with 21.9% (14/64) and *S.* Typhimurium with 9.4% (6/64). The findings related to antibiotic resistance genes revealed that the most frequently determined gene was *sul1* with approximately 58%, while the *blaTEM* gene was detected at the lowest proportion with 20%, among *Salmonella* isolates.

**Conclusions:**

The results indicated that *Salmonella* infections constitute a potential risk for chicken flocks in the country and that genotypic resistance rates against various antibiotics should draw particular attention in terms of both human and animal health.

## Background

*Salmonella* has been described as one of the most common foodborne pathogens worldwide leading to outbreaks of gastroenteritis in humans [1]. In epidemiological studies, food supplies of animal origin, in particular poultry, have been reported to be the main carriers of *Salmonella* infections to humans [2–4]. *Salmonella* agents often cause infections in humans by forming biofilms and contaminating food products by clustering on the surfaces of food and other materials [5]. *Salmonella* agents that cause infection in humans have been shown to be more common in poultry than in other animal species [6]. The presence of *Salmonella* in chicken meat and the other products may lead people to feel insecure about their consumption. Prevalence of *Salmonella* in chickens varies considerably between the countries. Field and abattoir based studies carried out in European countries revealed proportions ranging from 4 to 29% [7], while prevalence values ranging from 0.4 to 39% have been reported in different parts of Turkey [8–10].

Periodic identification of *Salmonella* serotypes circulating in poultry flocks is significant in terms of developing strategies for control and prevention of the disease and thereby for the trade of poultry products. Conventional detection of *Salmonella* agents is made by standard bacteriological culture method based on ISO 6579 protocols which takes 3 to 8 days. Although this method has been considered as the gold standard for the detection of *Salmonella*, owing to the time consumption researchers have preferred molecular based methods with high sensitivity and specificity such as polymerase chain reaction (PCR) in recent years [11–13]. Molecular studies on *Salmonella* have targeted different gene regions such as *invA* [14], *iroB* [15], *spiC* [16], *pipD* [17] and *int1* [18], and the results showed that the target gene region significantly affects the identification rates. The *invA* gene plays an important role in virulence of *Salmonella* agents and invasion into host cells [19]. In addition, the presence of the *invA* gene in all *Salmonella* species has led to its use as a gold marker in molecular based diagnostic studies to date [20].

Antibiotic resistance, which has become a global problem due to unconscious and uncontrolled use, makes treatment and control of *Salmonella* infections difficult as well as other bacterial diseases. In order to investigate antibiotic resistance levels, both genotypic methods that demonstrate the presence of genes causing resistance, and phenotypic methods such as disk diffusion method are used. It is known that multidrug resistance (MDR) in *Salmonella* strains is of zoonotic origin and thus can be transferred from animals to humans through consumption of contaminated products [21, 22]. Therefore, the determination of antibiotic resistance profiles in field isolates is important for revising the relevant legislations and developing more effective national strategies for preventing antibiotic resistance.

The aim of this study was to obtain quantitative data about the frequency, genotypic characterization and antibiotic resistance profiling of *Salmonella* agents in chicken flocks located in Elazig and the surrounding area.

## Results

*Salmonella* suspected colonies were determined in 84.4% (65/77) of the sock swab and internal organ samples which were subjected to isolation steps by ISO 6579:2002/A1:2007 method. Isolation was made from 75.5% (37/49) of the sock swabs collected in commercial poultry flocks, and from the internal organ samples of all (*n* = 28) necropsied animals belonging to small family enterprises. As a result of PCR analysis combined with a pair of primers specific to *invA* gene, 64 isolates produced amplification products at the molecular size of approximately 796 bp which is considered as indicative for the presence of *Salmonella* spp. One isolate obtained from the sock swab collected in a commercial layer flock could not be confirmed as *Salmonella* spp. in the PCR. Thus, the presence of *Salmonella* agents was confirmed in 36 (73.5%) of 49 poultry flocks (28 broiler and 8 layer) where sock swab samples were collected. In addition, all the isolates obtained from necropsied animals were found to be positive for *Salmonella* spp. by PCR (Table 1).

**Table 1:** Genotypic characterization of *Salmonella* isolates obtained from chicken samples

Sample Type	Number of *invA*-PCR Positive Samples	mPCR Findings			
		*S.* Typhimurium (%)	*S.* Enteritidis (%)	*S.* Infantis (%)	*Salmonella* spp. (%)
Sock Swabs					
Broiler (*n* = 39)	28	6 (21.4)	–	9 (32.1)	13 (46.4)
Layer (*n* = 10)	8	–	–	8 (100)	–
Internal Organs	28	–	14 (50)	–	14 (50)
Total	64	6 (9.4)	14 (21.9)	17 (26.6)	27 (42.2)

In the mPCR analysis of DNA samples extracted from 64 *Salmonella* spp. isolates, *S.* Infantis, *S.* Enteritidis and *S.* Typhimurium were identified in 17 (26.6%), 14 (21.9%) and 6 (9.4%) of the isolates, respectively. The difference between the identification proportions of *Salmonella* serotypes was statistically significant (*p* < 0.05). The remaining 27 *Salmonella* isolates could not be typed with the serotype-specific primers employed in this study. All the *Salmonella* isolates obtained from layer flocks were identified as *S.* Infantis, while *S.* Enteritidis could not be detected in any of the commercial flocks. The mPCR results of the isolates obtained from the chicken samples were presented in Table 1.

In order to determine the presence of antibiotic resistance genes, *Salmonella* isolates obtained in the present study were subjected to mPCR combined with primer pairs specific to resistance genes of four different antibiotics. All the *S.* Infantis isolates were found to harbor the *tetA* and *sul1* genes encoding tetracycline and trimethoprim-sulfamethoxazole resistance respectively, while only two contained resistance gene specific to ampicillin. It was determined that in two of *S.* Typhimurium isolates, genes encoding ampicillin and trimethoprim/sulfamethoxazole resistance were present, whereas four of them comprised the resistance gene specific to ampicillin alone. On the other hand, only one *S*. Enteritidis isolate was determined to contain the resistant genes specific to ampicillin, tetracycline and trimethoprim/sulfamethoxazole antibiotics, the rest of the isolates belonging to this serotype were not positive for the resistance genes tested in this study. Of the 27 *Salmonella* spp. isolates which could not be characterized with the serotype specific primers here, 17 were detected to harbor the resistance gene encoding trimethoprim/sulfamethoxazole, and four were positive for the ampicillin and tetracycline resistance genes (Table 2). However, none of the isolates obtained in the current study were determined to possess the *cat1* gene encoding chloramphenicol resistance. Overall evaluation of the antibiotic resistance profiling findings revealed that the most frequently determined gene was *sul1* encoding trimethoprim/sulfamethoxazole resistance with approximately 58%, while the *blaTEM* gene encoding ampicillin resistance was detected at the lowest proportion with 20%, among *Salmonella* isolates. The difference between these proportions was statistically significant (*p* < 0.05).

**Table 2:** mPCR results of antibiotic resistance profiling of *Salmonella* isolates obtained from chicken samples

Antibiotics	mPCR results of Salmonella isolates				
	*Salmonella* Typhimurium	*Salmonella* Enteritidis	*Salmonella* Infantis	*Salmonella* spp.	Total (%)
Tetracycline	–	1	17	4	22(34.4)
Trimethoprim/Sulfamethoxazole	2	1	17	17	37(57.8)
Ampicillin	6	1	2	4	13(20.3)
Chloramphenicol	–	–	–	–	–

DNA samples belonging to 13 randomly selected isolates among *Salmonella* spp. isolates which could not be typed with the specific primers used in the present study were subjected to sequence analysis. As a result of BLAST analysis, high similarity (99–100%) was detected between the partially sequenced isolates and *Salmonella enterica subsp. enterica* serovar Mbandaka strain CFSAN076213 (GenBank Accession Number: CP033343.1).

## Discussion

The expenses due to treatment and prevention of *Salmonella* infections in both poultry and humans impose great economic burden all over the world. For instance, the annual cost for the treatment of poultry originated *Salmonella* infections in the USA has been calculated to be as much as 14 billion dollars [23]. It is therefore an urge to develop effective control, eradication and prevention strategies toward *Salmonella* infections in both poultry and humans. In this respect, large scaled epidemiological investigations of diseases caused by *Salmonella* may provide useful data. From this point of view, this study was carried out to obtain quantitative data about the presence and frequency of Salmonella species circulation in chicken flocks in Turkey. Also, considering the fact that antibiotic resistance has become a global concern due unconscious and uncontrolled use which makes treatment and control of bacterial infection difficult, genotypic resistance profiling of the field isolates against various commonly used antibiotics was investigated in the present study.

Many studies have been conducted to reveal the presence and distribution of *Salmonella* species in chicken, worldwide and the prevalence rates ranging from 4 to 92% have been reported [24, 25]. On the other hand, field and abattoir based studies carried out different regions of Turkey have declared the proportions ranging from 0.4 to 39% [8–10]. In the analysis of sock swabs and internal organ samples collected from the total of 77 chicken flocks in the present study, *Salmonella* spp. identification was confirmed by PCR in 64 which corresponded to 83.1% overall. Many parameters such as geographical location, prevention/control and biosafety measures of the flocks, breeding conditions (cage/ground), flock management styles, sample size, sample type (drag swab, bedding, feces, internal organ, cloacal swab etc), sampling season and isolation and identification methods may be responsible in the occurrence of remarkably different isolation rates.

Sample type is considered as one of the significant parameters that play role in obtaining remarkable different results in terms of the frequency of *Salmonella*. In this study, sock swabs was preferred to collect fecal samples representing at least 20% of the flock area instead of collecting individual samples, considering its advantages such as being more practical, representing the whole flock, saving labor and time in addition to enhancing isolation chance and rate. In fact, the isolation rate obtained from the fecal samples in this study was remarkably high (73.5%) when compared with the report (7%) of Aksakal (2003) who examined cloacal swab samples collected from chickens in Van province [26]. This difference is not surprising because it has previously been reported that isolation chance of *Salmonella* from cloacal swabs might be decreased due to intermittent shedding in feces [27]. Similarly, Berghaus et al. [28] obtained 90% isolation rate from fecal samples collected via boot swab method which is similar to that used in the current study [28]. Also, in a study carried out in Spain, it was showed that while the isolation rate of *Salmonella* was 4% in cloacal swab samples, remarkably high percentage (92%) was obtained in the examination of feces samples collected using a sterile tongue depressor [24]. These studies put forward that sample collection method has a direct effect on the isolation rate of *Salmonella*.

An increase of 1 °C in the global temperature has been reported to enhance incidence of Salmonella which suggested that there may be a correlation between temperature and incidence of the disease [29]. In this study, the collection of fecal samples in July and August in which the temperature is very high may play a role in obtaining high isolation rate. Likewise, over 50% isolation rate has been reported in a field study conducted in Adana province which has one of the highest annual temperature in Turkey [10].

In most of the studies carried out in chickens all over the world, *S.* Enteritidis and *S.* Infantis have been reported to be more commonly identified serotypes than the others [20, 30–33]. Also, according to 2016 EFSA report, *S.* Infantis (37%) was the most commonly reported Salmonella serotype in broiler flocks, followed by *S.* Enteritidis (14%) [34]. In Turkey, previous studies based on conventional serotyping reported varied results in terms of the frequency of Salmonella agents depending on the study region and sample type but, overall evaluation of these studies showed that most commonly identified serotypes in chicken flocks were *S.* Enteritidis, *S.* Typhimurium and *S.* Infantis [9, 10, 26, 35–39]. In the present study, mPCR analysis of *Salmonella* sp. isolates revealed that *S.* Infantis, (26.6%) and *S.* Enteritidis (21.9%) were the most prevalent species followed by *S.* Typhimurium (9.4%). Although *S.* Infantis and *S.* Enteritidis isolation rates were close to each other, *S.* Infantis was found only in sock swab samples while *S.* Enteritidis in internal organ samples. The high isolation of *S*. Enteritidis from internal organs may be due to the fact that it is more invasive than other paratyphoid agents. On the other hand, *S.* Infantis has been shown to act as asymptomatic carrier in chickens and contaminate the environment as well as foods [40, 41]. Therefore, detection of *S*. Infantis as the most common serotype in this study was considered as an expected result. There are studies reporting the presence of other *Salmonella* species (*S.* Kentucky, *S.* Hadar, *S.* Liverpool, etc.) in poultry feces and internal organs [39]. The serotypes mentioned above were not included in this study because of the budget constraint, while *S.* Pullorum and *S*. Gallinarum were subject to the permission of the Food and Control General Directorate of Agriculture Ministry. However, partial sequence analysis of 13 randomly selected isolates that could not be typed in this study, showed very close homology with the strain of *S.* Mbandaka CFSAN076213 in the GeneBank.

The widespread use of antibiotics for promoting growth and prophylactic purpose in poultry flocks has led to bacterial resistance to antimicrobial agents [42]. Therefore, determination of resistance of *Salmonella* agents circulating in poultry farms to antibiotics commonly used in the market is important in terms of developing more effective treatment and control strategies. Many studies have been conducted to determine antibiotic resistance profiles of *Salmonella* agents in chickens worldwide, and resistance against many different antibiotics has been reported in chicken originated field isolates [4, 31, 32, 43]. According to EFSA report in 2016, among 2288 *Salmonella* isolates obtained from chicken flocks in the European Union, the highest resistance (between 40 and 50%) was detected against nalidixic acid, sulfamethoxazole and tetracycline antibiotics, whereas moderate and low resistance was noted against ampicillin (19%) and chloramphenicol (4%), respectively. When EFSA data were evaluated at serotype level, *S.* Infantis isolates had high resistance to nalidixic acid, sulfamethoxazole and tetracycline (over 80%), whereas the highest resistance among S. Enteritidis isolates were determined against nalidixic acid with 23%. On the other hand, high levels of resistance to tetracycline and ampicillin have been reported in *S.* Thyphimurium isolates obtained from laying hens [34]. Antibiotic resistance status of *Salmonella* species has been studied in Turkey as well, mostly based on phenotypical methods. In a number of studies carried out in different parts of Turkey, *Salmonella* isolates obtained from samples collected in both chicken flocks and slaughterhouses were reported to show the highest resistance against ampicillin [9, 10, 26, 35, 41]. The only molecular based study carried out in Sanliurfa province of Turkey so far, has reported that *S.* Infantis isolates obtained from various food sources showed high resistance against tetracycline and sulfonamide [44].

Among the 64 field isolates obtained in this study, the *sul1* gene was determined at the highest proportion with 57.8%, which was followed by *tetA* with 34.4% and *blaTEM* with 20.3%. On the other hand, none of the isolates were found to harbor *cat1* gene. When the antibiotic resistance profiles were considered at serotype level, all the *S.* Infantis isolates were detected to contain resistance genes encoding tetracycline and trimethoprim- sulfamethoxazole, whereas *S*. Typhimurium isolates were positive for the ampicillin resistance gene. When the genotypic resistance rates obtained in this study were evaluated in general, there was a similarity with the 2016 report of EFSA [34], but the presence of resistance gene specific to ampicillin was lower than the previous studies conducted in different regions of Turkey. Although many studies carried our elsewhere reported high resistance rate against nalidixic acid, it was not included in this study due to the fact that it is not used in the enterprises located in the study region. Also, other antibiotics tested in previous studies were beyond the scope of the present study due to budget limitations. Regional differences, antibiotic choice of the enterprise, methodology used for resistance detection and the number of isolates tested might be responsible for obtaining different resistance or susceptibility results against the same antibiotic. All in all, both literature data and the results obtained here suggest that antibiotic resistance in *Salmonella* species continue to pose a major problem in Turkey and elsewhere.

## Conclusion

In conclusion, the findings that *Salmonella* agents were isolated and identified from more than 70% of the flocks where sock swab samples were collected and that *S.* Infantis, *S.* Enteritidis and *S.* Typhimurium were detected to be the most dominant subtypes in the study region suggest that Salmonella infections constitute a potential risk for chicken flocks in the country. In addition, the results concerning genotypic resistance profiles of the isolates against various antibiotics in this and previous studies should draw particular attention in terms of both human and animal health. Antibiotic susceptibility testing before the selection of antibiotics for the treatment of *Salmonella* infections is therefore important in preventing unconscious and random use of antibiotics and minimizing the development of resistance in strains.

## Methods

### Sampling

Feces samples were collected from 49 different commercial chicken farms (39 broiler and 10 layer hens) in Elazig province and its surroundings located in eastern Turkey, between July and August 2018 with the help of sock swabs. The broiler flocks had the capacity ranging from 15.000 to 38.000 birds at the average age of 25 days, while the capacity of the layer flocks was between 3.000 and 10.000 birds at the average age of 32 weeks. All the commercial farms had ground type husbandry system. When collecting faecal samples via sock swabs, at least 20% of the flock area were considered as representative of the farm. In addition, internal organ (liver, spleen and cecum) samples taken at necropsy of 28 *Salmonella* suspected chickens belonging to small family enterprises in the region were included in the study. These animals were dispatched from the local family farms where few deaths have been reported. The animal capacity of the family enterprises showed a wide range from 12 to 2500 birds at the age of > 20 weeks. Fecal and internal organ samples were transported under aseptic conditions and cold chain to the laboratories of Department of Veterinary Microbiology, Firat University and, were examined for *Salmonella*.

### Isolation and identification of Salmonella

Isolation and identification of *Salmonella* species were performed according to ISO 6579: 2002/ Amd 1:2007 standard method [45]. For this purpose, inoculation steps to pre-enrichment, selective enrichment and selective-differential culture media were carried out. Each of the sock swabs and the internal organ samples (pooled to be 25 g) were homogenized with 250 ml sterile Buffered Peptone Water (BPW, 10%) and incubated at 37 °C for 18–24 h. For selective enrichment, 0.1 ml from the second day culture following pre-enrichment was transferred to two tubes, one containing 10 ml Rappaport-Vassiliadis Soy Broth (RVS) and the other containing 10 ml Muller Kauffmann Tetrathionate-Novobiocin Broth, and the tubes were incubated at 42 °C for 18–24 h. On the third day after incubation, a loopful of culture was inoculated with streaking plate method onto Brilliant Green Agar (BGA), Xylose Lysine Deoxycholate (XLD) Agar and MacConkey Agar for isolation and was incubated at 37 °C for 24–48 h. *Salmonella* suspected colonies (red with black centers in XLD agar, pink in BGA and colorless-transparent in MacConkey Agar) were purified in Nutrient Agar and were stored at − 20 °C in Nutrient Broth containing 20% glycerol for molecular analyses.

### DNA extraction

For the identification and molecular characterization of *Salmonella*, DNA extraction was carried out from cultures stored in Nutrient Broth. For this, suspected isolates were inoculated onto Nutrient Agar and were incubated at 37 °C for one day. Suspected colonies (7–8) were transferred to Eppendorf tubes containing 300 μl of distilled water and were homogenized. Each suspension was treated with 300 μl TNES buffer and 6 μl Proteinase K (20 mg/ml), and then inactivated at 56 °C for 2 h. After the suspension was boiled for 10 min, 400 μl phenol (saturated with Tris-HCl) was added and the mixture was shaken for 10 min followed by spinning at 11.600 g for 10 min. The upper phase was carefully transferred to another Eppendorf tube without touching phenol phase, and then DNA precipitation was performed. For this purpose, 30 μl of 3 M Na-acetate (0.1 volume) and 750 μl of pure alcohol (2.5 volume) were added to the suspension which was vortexed and kept at − 20 °C overnight. The suspension was then centrifuges at 11.600 g for 10 min, and the supernatant was carefully removed. The resulting pellet was washed with 70% ethanol and centrifuged at 11.600 g for 5 min. The final pellet was allowed to dry for 45 min and was suspended in 10 μl sterile distilled water. This suspension was used as target DNA in molecular analyzes.

### Polymerase chain reaction (PCR) and multiplex PCR (mPCR)

For the identification of *Salmonella* at genus level, PCR mixture was prepared in total volume of 25 μl containing 2.5 μl 10xPCR buffer, 2.5 μl 25 mM MgCl_2_, 2 μl dNTP Set, 0.25 μl 5 U / μl Taq DNA Polymerase enzyme, 1 μl of each of the genus-specific primer pair (Table 3) (20 pmol), 1 μl of target DNA and 14.75 μl of DNase-RNase free water.

**Table 3:** Primers used for the detection of *Salmonella* serotypes and antibiotic resistance genes in chicken isolates

Agent/ Antibiotics	Gene	Primer sequences	Fragment size (bp)	Literature
*Salmonella* sp.	*invA*-F	CGGTGGTTTTAAGCGTACTCTT	796	[14]
	*invA*-R	CGAATATGCTCCACAAGGTTA		
*Salmonella* Typhimurium	*SalfliC*-F	CCCCGCTTACAGGTGGACTAC	433	[20]
	*SalfliC*-R	AGCGGGTTTTCGGTGGTTGT		
*Salmonella* Enteritidis	*SdfIII*-F	GCTGACTCACACAGGAAATCG	350	[46]
	*SdfIII*-R	TCTGATAAGACTGGGTTTCACT		
*Salmonella* Infantis	*FljB-*F	TTGCTTCAGCAGATGCTAAG	413	[47]
	*FljB-*R	CCACCTGCGCCAACGCT		
Ampicillin	*blaTEM*-F	CATTTCCGTGTCGCCCTTAT	793	[48]
	*blaTEM*-R	TCCATAGTTGCCTGACTCCC		
Tetracycline	*tetA*-F	GCTACATCCTGCTTGCCTTC	210	[49]
	*tetA*-R	CATAGATCGCCGTGAAGAGG		
Trimethoprim-Sulfamethoxazole	*sul1*-F	TCACCGAGGACTCCTTCTTC	316	[48]
	*sul1*-R	AATATCGGGATAGAGCGCAG		
Chloramphenicol	*cat1*-F	CTTGTCGCCTTGCGTATAAT	508	[50]
	*cat1*-R	ATCCCAATGGCATCGTAAAG		

In order to identify *Salmonella* isolates at species level and to determine resistance status to various antibiotics, mPCR was performed. For this purpose, PCR mixtures in a total volume of 33 μl was prepared which contained 2.5 μl 10x PCR buffer, 2.5 μl 25 mM MgCl_2_, 2 μl dNTP Set, 0.25 μl 5 U / μl Taq DNA Polymerase enzyme, 1 μl (20 pmol) of each primer pairs specific for Salmonella species and antibiotic resistance genes (Table 3), 1 μl target DNA and 10.75 DNase-RNase free water.

The amplified PCR products were electrophoresed on a 1.5% agarose gel containing 10 μl of Ethidium Bromide solution, then examined under Ultraviolet transilluminator and the results were observed and photographed with Polaroid GelCam. A 100 bp DNA ladder was used to determine the molecular weight of the resulting bands. Following agarose gel electrophoresis, PCR products with the molecular sizes of approximately 796 bp, 433 bp, 413 bp and 350 bp were considered as indicative for *Salmonella* spp., *S.* Typhimurium, *S.* Infantis and *S.* Enteritidis, respectively. In addition, mPCR analysis for the presence of antibiotic resistance genes yielded products at the molecular sizes of approximately 793 bp for ampicillin, 508 bp for chloramphenicol, 316 bp for trimethoprim/sulfamethoxazole and 210 bp for tetracycline. In order to detect any possible contamination at any stage of the study, DNA samples belonging to *Salmonella* Typhimurium (NCTC- National Collection of Type Cultures- London, UK, 74), *Salmonella* Enteritidis ([NCTC, London, UK 12694) and *Salmonella* Infantis (Etlik Veterinary Control Research Center, Poultry Diseases Diagnostic Laboratory Collection) were used as positive controls, and DNase-RNase free water were used as negative control in both DNA extraction steps and PCR assays.

### Sequencing

DNA samples belonging to randomly selected 13 isolates, which were confirmed as *Salmonella* spp., but were not amplified by species specific primers employed in this study, were amplified with primers specific to *invA* gene and sent to Istanbul Pendik Veterinary Control Institute for partial sequence analysis in ABI 3130 XL genetic analyser (USA).

### Statistical analysis

The differences between the identification rates of *Salmonella* species and the proportion of resistance genes to antibiotics included in the study were evaluated by chi square (χ2) test and probability values of *P* < 0.05 were considered statistically significant.

## Data Availability

The datasets used and/or analysed during the current study are available from the corresponding author on reasonable request.

## Abbreviations

ISO: International Organization for Standardization
PCR: Polymerase chain reaction
mPCR: Multiplex polymerase chain reaction
EFSA: European Food Safety Authority
